# Supplementary material for: An open-source framework for large-scale, flexible evaluation of biomedical text mining systems
Source: J Biomed Discov Collab. 2008 Jan 29;3:1. doi: 10.1186/1747-5333-3-1 (PMC2276192; doi:10.1186/1747-5333-3-1)
Supplement: Additional file 1 — Evaluation platform source code. The additional file contains the software evaluation framework discussed in this paper. The most current release can be downloaded from SourceForge.net [1]. See the accompanying README file for instructions on installation and use. Also included in the distribution is a collection of UIMA wrappers for some commonly used BioNLP tools and annotated corpora. [file 1747-5333-3-1-S1.zip › bionlp_uima_repository-1.0.1/docs/api/src-html/edu/uchsc/ccp/util/external/lingpipe/LingPipe_Util.html]

```
001    package edu.uchsc.ccp.util.external.lingpipe;
002    
003    import java.io.File;
004    import java.io.FileInputStream;
005    import java.io.FileOutputStream;
006    import java.io.IOException;
007    import java.io.ObjectInputStream;
008    import java.io.ObjectOutputStream;
009    import java.io.PrintStream;
010    import java.io.StringReader;
011    import java.util.ArrayList;
012    import java.util.HashMap;
013    import java.util.Iterator;
014    import java.util.List;
015    import java.util.Map;
016    import java.util.Set;
017    import java.util.regex.Matcher;
018    import java.util.regex.Pattern;
019    
020    import org.xml.sax.Attributes;
021    import org.xml.sax.InputSource;
022    import org.xml.sax.SAXException;
023    import org.xml.sax.helpers.DefaultHandler;
024    
025    import com.aliasi.chunk.Chunk;
026    import com.aliasi.chunk.ChunkAndCharSeq;
027    import com.aliasi.chunk.Chunker;
028    import com.aliasi.chunk.Chunking;
029    import com.aliasi.classify.PrecisionRecallEvaluation;
030    import com.aliasi.corpus.ChunkHandler;
031    import com.aliasi.corpus.Parser;
032    import com.aliasi.corpus.parsers.GeniaEntityChunkParser;
033    import com.aliasi.corpus.parsers.GeniaPosParser;
034    import com.aliasi.corpus.parsers.GeniaSentenceParser;
035    import com.aliasi.corpus.parsers.MedPostPosParser;
036    import com.aliasi.hmm.HiddenMarkovModel;
037    import com.aliasi.hmm.HmmCharLmEstimator;
038    import com.aliasi.hmm.HmmDecoder;
039    import com.aliasi.io.FileExtensionFilter;
040    import com.aliasi.sentences.MedlineSentenceModel;
041    import com.aliasi.sentences.SentenceChunker;
042    import com.aliasi.sentences.SentenceEvaluation;
043    import com.aliasi.sentences.SentenceEvaluator;
044    import com.aliasi.sentences.SentenceModel;
045    import com.aliasi.tokenizer.IndoEuropeanTokenizerFactory;
046    import com.aliasi.tokenizer.RegExTokenizerFactory;
047    import com.aliasi.tokenizer.Tokenizer;
048    import com.aliasi.tokenizer.TokenizerFactory;
049    import com.aliasi.util.AbstractExternalizable;
050    import com.aliasi.util.Streams;
051    
052    import edu.uchsc.ccp.util.nlp.GenericTextUtils;
053    import edu.uchsc.ccp.util.nlp.annotation.AnnotationSet;
054    import edu.uchsc.ccp.util.nlp.annotation.Annotation_Util;
055    import edu.uchsc.ccp.util.nlp.annotation.Annotator;
056    import edu.uchsc.ccp.util.nlp.annotation.TextAnnotation;
057    import edu.uchsc.ccp.util.nlp.mention.ClassMention;
058    import edu.uchsc.ccp.util.nlp.mention.ClassMentionTypes;
059    
060    public class LingPipe_Util {
061        private static TokenizerFactory TOKENIZER_FACTORY = new IndoEuropeanTokenizerFactory();
062    
063        private static final SentenceModel SENTENCE_MODEL = new MedlineSentenceModel();
064    
065        private static final SentenceChunker SENTENCE_CHUNKER = new SentenceChunker(TOKENIZER_FACTORY, SENTENCE_MODEL);
066    
067        private static HmmDecoder decoder = null;
068    
069        // corresponds with the CCP Annotation Database
070        private static final int annotatorID = 22;
071    
072        private static final boolean DEBUG = false;
073    
074        private static final Annotator annotator = new Annotator(new Integer(22), "", "LingPipe", "Alias-i");
075    
076        private static final AnnotationSet annotationSet = new AnnotationSet(-1, "Default Set","Default Set");
077        
078        private static Chunker entityChunker;
079    
080        private static String entityType;
081    
082        private static boolean posTaggerInitialized = false;
083    
084        private static boolean entityTaggerInitialized = false;
085    
086        /**
087         * Use SentenceModel to find sentence boundaries in text LingPipe relevant code used by this method was taken from:
088         * http://alias-i.com/lingpipe/demos/tutorial/sentences/src/SentenceChunkerDemo.java
089         * 
090         * @param inputText
091         */
092        public static List<TextAnnotation> findSentences(String inputText, String documentID) {
093            // this method will return a list of TextAnnotations; one per sentence
094            List<TextAnnotation> annotations = new ArrayList<TextAnnotation>();
095    
096            Chunking chunking = SENTENCE_CHUNKER.chunk(inputText.toCharArray(), 0, inputText.length());
097            Set sentences = chunking.chunkSet();
098            if (sentences.size() < 1) {
099                if (inputText.trim().length() > 0) {
100                    System.out.println("WARNING -- LingPipe_Util: No sentence chunks found for input text: \"" + inputText + "\"");
101                }
102                return annotations;
103            }
104            // String slice = chunking.charSequence().toString();
105            // int i = 1;
106            for (Iterator it = sentences.iterator(); it.hasNext();) {
107                Chunk sentence = (Chunk) it.next();
108                int start = sentence.start();
109                int end = sentence.end();
110    
111                TextAnnotation ta = new TextAnnotation();
112                ta.setAnnotator(annotator);
113                ta.setDocumentID(documentID);
114                ta.setCoveredText(inputText.substring(start, end));
115                    ta.setAnnotationSpanEnd(end);
116                    ta.setAnnotationSpanStart(start);
117    
118                    ta.addAnnotationSet(annotationSet);
119                    
120                ClassMention cm = new ClassMention(ClassMentionTypes.SENTENCE);
121                ta.setClassMention(cm);
122    
123                annotations.add(ta);
124    
125                // System.out.println("SENTENCE " + (i++) + ":");
126                // System.out.println(slice.substring(start, end));
127            }
128            return annotations;
129        }
130    
131        /**
132         * Much of this code was taken from: http://alias-i.com/lingpipe/demos/tutorial/posTags/src/TrainMedPost.java
133         * 
134         */
135        public static void trainMedPostTagger(String medPostCorpusLocation, String outputModelFileLocation) throws IOException {
136    
137            // language model parameters for HMM emissions
138            final int N_GRAM = 8;
139            final int NUM_CHARS = 256;
140            final double LAMBDA_FACTOR = 8.0;
141    
142            // set up parser with estimator as handler
143            HmmCharLmEstimator estimator = new HmmCharLmEstimator(N_GRAM, NUM_CHARS, LAMBDA_FACTOR);
144            Parser parser = new MedPostPosParser();
145            parser.setHandler(estimator);
146    
147            // train on files in data directory ending in "ioc"
148            File dataDir = new File(medPostCorpusLocation);
149            File[] files = dataDir.listFiles(new FileExtensionFilter("ioc"));
150            for (int i = 0; i < files.length; ++i) {
151                System.out.println("Training file=" + files[i]);
152                parser.parse(files[i]);
153            }
154    
155            // write output to file
156            File modelFile = new File(outputModelFileLocation);
157            FileOutputStream fileOut = new FileOutputStream(modelFile);
158            ObjectOutputStream objOut = new ObjectOutputStream(fileOut);
159            estimator.compileTo(objOut);
160            Streams.closeOutputStream(objOut);
161        }
162    
163        /**
164         * 
165         * @param GENIACorpusLocation
166         * @param outputModelFileLocation
167         * @throws IOException
168         */
169        public static void trainGENIATagger(String GENIACorpusLocation, String outputModelFileLocation) throws IOException {
170            // language model parameters for HMM emissions
171            final int N_GRAM = 8;
172            final int NUM_CHARS = 256;
173            final double LAMBDA_FACTOR = 8.0;
174    
175            // set up parser with estimator as handler
176            HmmCharLmEstimator estimator = new HmmCharLmEstimator(N_GRAM, NUM_CHARS, LAMBDA_FACTOR);
177            Parser parser = new GeniaPosParser();
178            parser.setHandler(estimator);
179    
180            // train on files in data directory ending in "ioc"
181            File geniaCorpusFile = new File(GENIACorpusLocation);
182            System.out.println("Training file=" + geniaCorpusFile);
183            parser.parse(geniaCorpusFile);
184    
185            // write output to file
186            File modelFile = new File(outputModelFileLocation);
187            FileOutputStream fileOut = new FileOutputStream(modelFile);
188            ObjectOutputStream objOut = new ObjectOutputStream(fileOut);
189            estimator.compileTo(objOut);
190            Streams.closeOutputStream(objOut);
191        }
192    
193        /**
194         * Much of this code was taken from: http://alias-i.com/lingpipe/demos/tutorial/posTags/src/RunMedPost.java NOTE:
195         * initializeTaggingSystem() must be called before this method.
196         * 
197         * @param medPostModelFile
198         * @param inputText
199         */
200        public static ArrayList<TextAnnotation> runPOSTagger(String inputText, String documentID) {
201            if (posTaggerInitialized) {
202                ArrayList<TextAnnotation> posAnnotations = new ArrayList<TextAnnotation>();
203                char[] cs = inputText.toCharArray();
204                Tokenizer tokenizer = TOKENIZER_FACTORY.tokenizer(cs, 0, cs.length);
205                try {
206                String[] tokens = tokenizer.tokenize();
207                String[] tags = decoder.firstBest(tokens);
208                posAnnotations = getTagAnnotations(tokens, tags, inputText, documentID);
209                } catch (Exception e) {
210                    warn("Caught exception while tokenizing text: DOCID=" + documentID + " TEXT=" + inputText);
211                }
212                return posAnnotations;
213            } else {
214                System.err.println("LingPipe POS Tagger has not been initialized. Please call initializePOSTaggingSystem() prior to tagging text.");
215                return null;
216            }
217        }
218    
219        /**
220         * Ready the system for POS tagging.
221         * 
222         */
223        public static void initializePOSTaggingSystem(String modelFile) throws ClassNotFoundException, IOException {
224            TOKENIZER_FACTORY = new RegExTokenizerFactory("(-|'|\\d|\\p{L})+|\\S");
225    
226            System.out.println("Reading model from file=" + modelFile);
227            FileInputStream fileIn = new FileInputStream(modelFile);
228            ObjectInputStream objIn = new ObjectInputStream(fileIn);
229            HiddenMarkovModel hmm = (HiddenMarkovModel) objIn.readObject();
230            Streams.closeInputStream(objIn);
231            decoder = new HmmDecoder(hmm);
232    
233            posTaggerInitialized = true;
234        }
235    
236        private static ArrayList<TextAnnotation> getTagAnnotations(String[] tokens, String[] tags, String inputText, String documentID) {
237            ArrayList<TextAnnotation> returnAnnotations = new ArrayList<TextAnnotation>();
238            /* originalIndex keeps track of where you are in the original text */
239            int originalIndex = 0;
240            Pattern origTextPattern;
241            Matcher origTextMatcher;
242    
243            /* cycle through each token, find it's span in the original text, and create a new TextAnnotation */
244            for (int i = 0; i < tokens.length; i++) {
245                TextAnnotation ta = new TextAnnotation();
246                ta.setAnnotator(annotator);
247                ta.setDocumentID(documentID);
248    
249                if (DEBUG) {
250                    System.out.println(" Looking for: " + tokens[i] + "_" + tags[i] + "\tstarting at originalIndex: " + originalIndex);
251                }
252    
253                /* look for the tagged text in the input text to get the correct span */
254                origTextPattern = Pattern.compile(GenericTextUtils.escapeCharacterForRegEx(tokens[i]));
255                origTextMatcher = origTextPattern.matcher(inputText);
256    
257                if (origTextMatcher.find(originalIndex)) {
258                        /* set the span end */
259                        ta.setAnnotationSpanEnd(origTextMatcher.end());
260                        /* set the span beginning */
261                        ta.setAnnotationSpanStart(origTextMatcher.start());
262    
263                    /* set the covered text from the original (untagged) text */
264                    ta.setCoveredText(origTextMatcher.group());
265    
266                    /* set the class mention for the annotation to be a token, with a slot for POS (stem and lemma are set to null) */
267                    ta.setClassMention(Annotation_Util.createTokenMention(tags[i], null, null, i));
268    
269                    /* check to make sure span appear legitimate */
270                    if ((ta.getAnnotationSpanEnd() - ta.getAnnotationSpanStart()) != ta.getCoveredText().length()) {
271                        System.err.println("Annotation span lengths do not match. " + (ta.getAnnotationSpanEnd() - ta.getAnnotationSpanStart())
272                                + " != " + ta.getCoveredText().length());
273                        ta.printAnnotation(System.err);
274                    }
275                    /* update originalIndex so that next search starts at end of previous */
276                    originalIndex = ta.getAnnotationSpanEnd();
277                    returnAnnotations.add(ta);
278                } else {
279                    /* something is wrong because it should have found the text */
280                    System.err.println("Error, could not find original text: " + tokens[i] + " starting at: " + originalIndex + " in : "
281                            + inputText);
282                }
283            }
284            return returnAnnotations;
285        }
286    
287        public static int getAnnotatorID() {
288            return annotatorID;
289        }
290    
291        /**
292         * Evaluate the LingPipe Sentence detector Taken from:
293         * http://alias-i.com/lingpipe/demos/tutorial/sentences/src/SentenceModelEvaluator.java
294         * 
295         */
296        public static void evaluateSentenceDetector(String evaluationCorpusLocation, String errorFilePath) throws SAXException, IOException {
297            TokenizerFactory tokenizerFactory = new IndoEuropeanTokenizerFactory();
298            SentenceModel sentenceModel = new MedlineSentenceModel();
299            SentenceChunker sentenceChunker = new SentenceChunker(tokenizerFactory, sentenceModel);
300            SentenceEvaluator sentenceEvaluator = new SentenceEvaluator(sentenceChunker);
301    
302            GeniaSentenceParser parser = new GeniaSentenceParser(sentenceEvaluator);
303            // parser.setHandler(sentenceEvaluator);
304    
305            File inFile = new File(evaluationCorpusLocation);
306            parser.parse(inFile);
307    
308            SentenceEvaluation sentenceEvaluation = sentenceEvaluator.evaluation();
309    
310            PrecisionRecallEvaluation chunkingStats = sentenceEvaluation.chunkingEvaluation().precisionRecallEvaluation();
311            System.out.println("Chunking Evaluation statistics");
312            System.out.println(chunkingStats.toString());
313            System.out.println("");
314    
315            PrecisionRecallEvaluation endBoundaryStats = sentenceEvaluation.endBoundaryEvaluation();
316            System.out.println("Sentence Evaluation end boundary statistics");
317            System.out.println(endBoundaryStats.toString());
318    
319            // print error diagnostics to files
320            int i = 0;
321            Set falseNegatives = sentenceEvaluation.falseNegativeEndBoundaries();
322            PrintStream falseNegOut = new PrintStream(new FileOutputStream(errorFilePath + "EvaluatorFalseNegatives.txt"));
323            for (Iterator it = falseNegatives.iterator(); it.hasNext(); ++i) {
324                ChunkAndCharSeq sentence = (ChunkAndCharSeq) it.next();
325                falseNegOut.println(i + ". " + sentence.spanEndContext(34));
326            }
327            falseNegOut.close();
328            int j = 0;
329            Set falsePositives = sentenceEvaluation.falsePositiveEndBoundaries();
330            PrintStream falsePosOut = new PrintStream(new FileOutputStream(errorFilePath + "EvaluatorFalsePositives.txt"));
331            for (Iterator it = falsePositives.iterator(); it.hasNext(); ++j) {
332                ChunkAndCharSeq sentence = (ChunkAndCharSeq) it.next();
333                falsePosOut.println(j + ". " + sentence.spanEndContext(34));
334            }
335            falsePosOut.close();
336    
337        }
338    
339        /*
340         * 
341         * ref: ------------X-------------+------ text: See Spot run. Run spot run. (...) resp: --------------------------+------ pos:
342         * 11111111112222222222 pos: 012345668901234567890123456789
343         */
344    
345        /**
346         * Use with Lingpipe_3.0.0
347         */
348        public static void initializeEntityTaggingSystem(String modelFile, String entityType) throws IOException {
349            File entityModelFile = new File(modelFile);
350    //        IndoEuropeanTokenCategorizer tokenCategorizer = new IndoEuropeanTokenCategorizer();
351    //        double threshold = 16.0;
352    //        Decoder entityDecoder = new Decoder(entityModelFile, tokenCategorizer, threshold);
353    //        entityChunker = new NEChunker(TOKENIZER_FACTORY, entityDecoder);
354            try {
355                entityChunker = (Chunker) AbstractExternalizable.readObject(entityModelFile);
356            } catch (ClassNotFoundException e) {
357                e.printStackTrace();
358            }
359            LingPipe_Util.entityType = entityType;
360            entityTaggerInitialized = true;
361        }
362    
363        /**
364         * Use with Lingpipe_3.0.0
365         */
366        public static List<TextAnnotation> extractEntities(String inputText, String documentID) {
367            if (entityTaggerInitialized) {
368            List<TextAnnotation> entityAnnotations = new ArrayList<TextAnnotation>();
369    
370            Chunking chunking = entityChunker.chunk(inputText.toCharArray(), 0, inputText.length());
371            Set mentions = chunking.chunkSet();
372            for (Iterator it = mentions.iterator(); it.hasNext();) {
373                Chunk mention = (Chunk) it.next();
374                int start = mention.start();
375                int end = mention.end();
376                
377                TextAnnotation ta = new TextAnnotation();
378                ta.setAnnotator(annotator);
379                ta.setDocumentID(documentID);
380                ta.setCoveredText(inputText.substring(start, end));
381                    ta.setAnnotationSpanEnd(end);
382                    ta.setAnnotationSpanStart(start);
383    
384                ClassMention cm;
385                if (entityType != null) {
386                    cm = new ClassMention(entityType);
387                } else {
388                    cm = new ClassMention(mention.type());
389                }
390                ta.setClassMention(cm);
391    
392                entityAnnotations.add(ta);
393    
394            }
395    
396            return entityAnnotations;
397            } else {
398                System.err.println("LingPipe Entity tagger not initialized. Please call initializeEntityTaggingSystem() prior to tagging entities.");
399                return null;
400            }
401        }
402    
403        
404        
405    //    /**
406    //     * Use with Lingpipe_2.1.1
407    //     */
408    //    public static void initializeEntityTaggingSystem(String modelFile, String entityType) throws IOException {
409    //        File entityModelFile = new File(modelFile);
410    //        IndoEuropeanTokenCategorizer tokenCategorizer = new IndoEuropeanTokenCategorizer();
411    //        double threshold = 16.0;
412    //        Decoder entityDecoder = new Decoder(entityModelFile, tokenCategorizer, threshold);
413    //        entityChunker = new NEChunker(TOKENIZER_FACTORY, entityDecoder);
414    //        LingPipe_Util.entityType = entityType;
415    //        entityTaggerInitialized = true;
416    //    }
417    //
418    //    /**
419    //     * Use with Lingpipe_2.1.1
420    //     */
421    //    public static List<TextAnnotation> extractEntities(String inputText, String documentID) {
422    //        if (entityTaggerInitialized) {
423    //        List<TextAnnotation> entityAnnotations = new ArrayList<TextAnnotation>();
424    //
425    //        Chunking chunking = entityChunker.chunk(inputText.toCharArray(), 0, inputText.length());
426    //        Set mentions = chunking.chunkSet();
427    //        for (Iterator it = mentions.iterator(); it.hasNext();) {
428    //            Chunk mention = (Chunk) it.next();
429    //            int start = mention.start();
430    //            int end = mention.end();
431    //            
432    //            TextAnnotation ta = new TextAnnotation();
433    //            ta.setAnnotator(annotator);
434    //            ta.setDocumentID(documentID);
435    //            ta.setCoveredText(inputText.substring(start, end));
436    //                ta.setAnnotationSpanEnd(end);
437    //                ta.setAnnotationSpanStart(start);
438    //
439    //            ClassMention cm = new ClassMention();
440    //            if (entityType != null) {
441    //                cm.setMentionName(entityType);
442    //            } else {
443    //                cm.setMentionName(mention.type());
444    //            }
445    //            ta.setClassMention(cm);
446    //
447    //            entityAnnotations.add(ta);
448    //
449    //        }
450    //
451    //        return entityAnnotations;
452    //        } else {
453    //            System.err.println("LingPipe Entity tagger not initialized. Please call initializeEntityTaggingSystem() prior to tagging entities.");
454    //            return null;
455    //        }
456    //    }
457    
458        // /**
459        // *
460        // * @param args
461        // * args[0]: MedPost Corpus Location<br>
462        // * args[1]: MedPost Model File<br>
463        // * args[2]: GENIA Corpus Location (tgz file)<br>
464        // * args[3]: GENIA Model File<br>
465        // * args[4]: method to call<br>
466        // * args[5]: text to tag<br>
467        // * args[6]: evaluation corpus file (e.g. GENIAcorpus3.02.xml) args[7]: evaluation error file path
468        // */
469        // public static void main(String[] args) {
470        // try {
471        // if (args[4].equals("trainMedPostTagger")) {
472        // LingPipe_Util.trainMedPostTagger(args[0], args[1]);
473        // } else if (args[4].equals("trainGENIATagger")) {
474        // LingPipe_Util.trainGENIATagger(args[2], args[3]);
475        // } else if (args[4].equals("runMedPostTagger")) {
476        // LingPipe_Util.initializePOSTaggingSystem(args[1]);
477        // ArrayList annotations = LingPipe_Util.runPOSTagger(args[5], "docID:1234");
478        // for (Iterator iter = annotations.iterator(); iter.hasNext();) {
479        // TextAnnotation ta = (TextAnnotation) iter.next();
480        // ta.printAnnotation(System.out);
481        // }
482        // } else if (args[4].equals("evaluateSentenceDetector")) {
483        // evaluateSentenceDetector(args[6], args[7]);
484        // } else if (args[4].equals("runGENIATagger")) {
485        //
486        // } else {
487        // System.out.println("Invalid method name parameter.");
488        // }
489        // } catch (Exception ioe) {
490        // ioe.printStackTrace();
491        // }
492        // }
493    
494        
495        
496        /****************************************/
497        /* GENIA CORPUS PARSING UTILITIES BELOW */
498        /****************************************/
499         
500        private static Map<String, List<String>> sentence2entitiesMap;
501    
502        private static List<String> sentences;
503    
504        /**
505         * parse input text that is in the GENIA (XML-embedded entity) format
506         * 
507         * @param geniaText
508         */
509        public static void parseGeniaText(String geniaText) throws SAXException, IOException {
510            /* initialize the storage for sentences and entities */
511            sentences = new ArrayList<String>();
512            sentence2entitiesMap = new HashMap<String, List<String>>();
513    
514            GeniaChunkHandler handler = new GeniaChunkHandler();
515    
516            StringReader sr = new StringReader(geniaText);
517    
518            GeniaEntityChunkParser gep = new GeniaEntityChunkParser(handler);
519            InputSource is = null;
520            is = new InputSource(sr);
521    
522            gep.parse(is);
523            
524            
525            System.out.println("GSPGSPGSPGSPGSPGSPGSPGSPGSPGSPGSPGSPGSPGSPGSPGSPGSPGSPGSPGSPGSPGSPGSPGSPGSPGSPGSPGSPGSPGSP");
526            handler = new GeniaChunkHandler();
527            GeniaSentenceParser gsp = new GeniaSentenceParser(handler);
528            sr = new StringReader(geniaText);
529            is = new InputSource(sr);
530            gsp.parse(is);
531            
532        }
533    
534        /*
535         * The GeniaChunkHandler class
536         */
537        private static class GeniaChunkHandler extends DefaultHandler implements ChunkHandler {
538    
539            public void handle(Chunking chunking) {
540                /* create a new sentence */
541                String sentence = chunking.charSequence().toString();
542                sentences.add(sentence);
543                
544                /* extract the entities for the new sentence */
545                List<String> entityList = new ArrayList<String>();
546                Set<Chunk> chunkSet = chunking.chunkSet();
547                for (Chunk chunk : chunkSet) {
548                    /* crop the output string at the @ symbol */
549                    String entityStr = chunk.toString();
550                    entityStr = entityStr.substring(0, entityStr.indexOf("@"));
551                    entityList.add(entityStr);
552                    System.out.println("CHUNK -- TYPE: " + chunk.type());
553                    System.out.println("CHUNK -- START: " + chunk.start());
554                    System.out.println("CHUNK -- END: " + chunk.end());
555                    System.out.println("CHUNK -- toString: " + chunk.toString());
556                }
557                
558                
559    
560                sentence2entitiesMap.put(sentence, entityList);
561    
562            }
563    
564            @Override
565            public void startElement(String uri, String localName, String qName, Attributes attributes) throws SAXException {
566               System.out.println("FOUND START ELEMENT: " + localName + " -- " + qName);
567                super.startElement(uri, localName, qName, attributes);
568            }
569        }
570    
571        /**
572         * Returns a list of sentences that were extracted from the previously parsed Genia input text
573         * 
574         * @return
575         */
576        public static List<String> getGeniaSentencesFromParse() {
577            return sentences;
578        }
579    
580        /**
581         * Returns a mapping between sentence text and entity sets from the previously parsed Genia input text. The entities take the form of
582         * "spanStart-spanEnd: classMentionName"
583         * 
584         * @return
585         */
586        public static Map<String, List<String>> getGeniaEntitiesFromParse() {
587            return sentence2entitiesMap;
588        }
589    
590        
591        public static void warn (String message) {
592            System.err.println("WARNING -- LingPipe_Util: " + message);
593        }
594        
595        
596        
597    }
```
